# Long‐Acting Naltrexone Restores Network Connectivity in Subjects With Comorbid Cannabis and Opioid Use Disorder

**DOI:** 10.1111/adb.70159

**Published:** 2026-05-17

**Authors:** Lindsey M. Brier, Daniel D. Langleben, Corinde E. Wiers, Zhenhao Shi

**Affiliations:** ^1^ Center for Studies of Addiction, Department of Psychiatry, Perelman School of Medicine University of Pennsylvania Philadelphia Pennsylvania USA; ^2^ Department of Radiology, Perelman School of Medicine University of Pennsylvania Philadelphia Pennsylvania USA

**Keywords:** alcohol use disorder, cannabis use disorder, cocaine use disorder, fMRI, functional connectivity, naltrexone

## Abstract

Comorbid substance use disorders (**SUDs**) are common but difficult to study due to the complex, interacting and overlapping mechanisms through which they affect brain networks. Many datasets collected to investigate a specific SUD include participants with comorbid SUDs. Although most studies treat comorbid SUDs as covariates of no interest, these covariates also contain untapped information. This is particularly relevant as cannabis use disorder (**CanUD**) has become increasingly prevalent and comorbid with other SUDs that have been more thoroughly studied. While pharmacotherapies have been established for multiple SUDs, none have been approved for CanUD, although naltrexone (**NTX**) has been associated with reduced use. Here, we conducted a retrospective secondary analysis of functional magnetic resonance imaging (**fMRI**) data from individuals with primary opioid use disorder (**OUD‐only**, *N* = 25 pre‐NTX, *N* = 20 on‐NTX) with comorbid CanUD (*N* = 10), alcohol use disorder (**AUD**, *N* = 6) or cocaine use disorder (**CocUD**, *N* = 7). All participants underwent imaging prior to receiving a therapeutic dose of long‐acting intramuscular NTX (Vivitrol), an approved treatment for OUD and AUD but not for CocUD, and again 2 weeks postadministration. At baseline, OUD individuals with comorbid CanUD, AUD or CocUD exhibited distinct functional connectivity (**FC**) alterations compared to those with OUD‐only. These differences were greater in younger participants and primarily involved the default mode network. Following NTX administration, FC differences between the comorbid CanUD and OUD‐only groups globally diminished. A similar FC response to NTX was observed in the comorbid AUD group, whereas little change in FC was observed in comorbid CocUD. These findings, combined with prior evidence that NTX reduces cannabis use by dampening reward, suggest NTX may hold promise as a treatment for CanUD.

## Graphical abstract

This study describes functional connectivity (FC) in subjects with opioid use disorder (OUD‐only), OUD+CanUD (cannabis use disorder), OUD+AUD (alcohol use disorder), and OUD+CocUD (cocaine use disorder) before and after naltrexone (NTX). All groups displayed hyper connectivity at baseline; however, on‐NTX, OUD+CanUD and OUD+AUD showed a decrease in hyper connectivity, while OUD+CocUD did not change. As NTX is an approved treatment for OUD and AUD, our analyses add to the growing literature investigating the utility of NTX in CanUD.

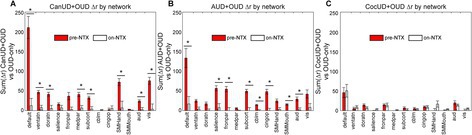

## 1 Introduction

Co‐occurring and comorbid substance use disorders (**SUDs**) are becoming the norm rather than the exception among individuals who misuse alcohol, cannabis, opioids or cocaine, as well as those with a psychiatric disorder [1]. The aetiology of these comorbidities is likely multifaceted, involving impure street manufacturing processes, social environments and norms surrounding use, self‐medication of symptoms or side effects from one substance with another, and attempts to maximize the psychoactive effects of any one substance [2]. A well‐known example is the concurrent use of alcohol and cocaine, which produces cocaethylene, a psychoactive metabolite that, like cocaine, inhibits the reuptake of dopamine but has a longer half‐life, resulting in prolonged and intensified intoxication. Some users also combine alcohol (a depressant) with cocaine (a stimulant) to mitigate the undesirable effects of either substance when taken alone [3]. However, for other substance combinations, the perceived benefits to users are less clearly understood.

Among patients with opioid use disorder (**OUD**), approximately 28% have comorbid cannabis use disorder (**CanUD**) [4]. The reasons for this co‐occurrence remain unclear, though contributing factors include cannabis' potential to reduce the severity of opioid withdrawal symptoms and the parallel rise of cannabis legalization and the opioid epidemic in the United States [5]. The phenomenology of OUD has evolved significantly since the 20th century era of injectable heroin abuse as heroin has largely been supplanted by illicitly manufactured fentanyl, a synthetic opioid many times more potent than heroin and the leading cause of overdose deaths in the past decade [6]. Medication for opioid use disorder (**MOUD**) such as methadone and buprenorphine is effective in aiding detoxification and maintaining sobriety in OUD and has been utilized more frequently and for longer in response to spreading opioid use [7, 8].

Simultaneously, cannabis use in the United States has steadily increased. As of 2022, 25% of individuals aged 12 or older reported cannabis use, with 19 million meeting DSM‐5 criteria for CanUD [5]. The legal cannabis industry is now valued at nearly $40 billion, with continued growth projected. This trend coincides with expanding state‐level legalization and a declining perception of risk among the public [9]. In response, dispensaries are producing cannabinoid products with higher purity and potency, the most popular containing the dually psychoactive and addictive delta‐9‐tetrahydrocannabinol (**THC**). Subjectively, reported benefits of THC usage include feelings of relaxation, pleasure and dissociation in the short term [5]. Objectively, research has indicated an increased incidence of cognitive and mood disorders, anxiety and psychosis in the long term [10, 11, 12, 13].

At the molecular level, prior works have begun to explore the neurobiological basis of OUD and CanUD comorbidity. Both opioid and cannabinoid (**CB**) receptors are G‐protein coupled and colocalize on presynaptic terminals in limbic, mesencephalon, brainstem and spinal cord regions [14]. The exact mechanism by which opioid and CB receptors interact is unknown; however, some studies suggest that mu‐opioid and CB1 receptors function together, as either heterodimers or allosteric modulators would [15]. Elsewhere in clinical studies, patients receiving both THC and opioid analgesics for treatment of chronic pain reported a 27% decrease in pain compared to opioids alone without any change in blood opioid concentration, concluding that THC augments the experience of opioids by some other mechanism [16].

Cognitive behavioural therapy and contingency management are effective individual therapies, whereas couples and family therapy are effective group‐based therapies for SUDs (including CanUD) [17]. However, there are currently no FDA‐approved medications for the treatment of CanUD. As mentioned above, multiple medication‐based treatments are FDA‐approved for OUD. Within the category of opioid agonist treatments are the MOUDs mentioned earlier, full opioid agonist methadone and partial opioid agonist buprenorphine. Currently, these medications are popular as they are initially used for detoxification from illicit opioid use and many patients elect to continue them as maintenance therapy [7, 8]. Although the recommended duration of MOUD is unknown, it is measured in years, with early discontinuation highly predictive of recurrence of use [18, 19]. The need for long‐term treatment and high risk of nonadherence led to the development of long‐acting injectable preparations. Buprenorphine and opioid antagonist naltrexone (**NTX**) are both medications that come in longer acting injectable forms that can help mitigate this burden by requiring a once‐a‐month shot for MOUD [20].

NTX is also an efficacious treatment for alcohol use disorder (**AUD**). Broadly, NTX inhibits B‐endorphin activity by blocking opioid receptors in the ventral tegmental area and nucleus accumbens, which leads to a decrease in dopamine release, translating to reduced reward associated with substance use and therefore lower levels of use [21]. Although NTX has been clinically proven to decrease levels of alcohol and opioid use, data have not supported NTX in the treatment of cocaine use disorder (**CocUD**) [22]. This may be due to cocaine producing a euphoric effect through dopamine reuptake blockade as opposed to opioid receptor modulation [23]. Although NTX is known to reduce craving and reward of various substances [24], its benefit for CanUD is uncertain. However, a clinical study supplying long‐acting NTX to cannabis users noticed a decrease in the number of days of use and overall perceived reward from using within 2 weeks of being on‐NTX. This finding was maintained during the post study follow up and postulates that NTX could have some utility in treating CanUD or helping cannabis users cut back on use in the same manner it is effective in OUD and AUD; however, further investigation is needed [25].

Functional magnetic resonance imaging (**fMRI**) is a non‐invasive modality that has the unique ability to explore neuroscientific phenomena at both the molecular and systems level [26, 27]. In addiction research, fMRI has been used to describe a degradation in functional network segregation in adults of all ages with OUD and AUD that scales with addiction severity and is similar to what would be expected in age‐related cognitive decline [28, 29]. In CanUD, most neuroimaging studies focus on adolescent subjects. In this population, there is evidence for THC‐induced altered hippocampal activity during memory encoding [30]. Also in adolescents, functional connectivity (**FC**) was noted to increase in the dorsal medial prefrontal cortex and decrease in the dorsal visual stream networks following acute THC consumption in chronic cannabis users [31], though no similar analysis has been done in adults. Functional readouts such as these are frequently used as biomarkers that track severity of illness as well as response to treatment and have been rigorously validated in disease processes such as Alzheimer's disease [32], stroke [33] and multiple sclerosis [34].

Here, we analysed resting state (**rs**) brain fMRI data that was collected in OUD patients before and after receiving NTX. Each participant had accompanying data on other substance use as well as comorbid SUDs, allowing us to separate the data into OUD with comorbid CanUD (**CanUD+OUD**), OUD with comorbid AUD (**AUD+OUD**) and OUD with comorbid CocUD (**CocUD+OUD**) compared to OUD‐only (to control for opioid use). We used the Power atlas to define 264 regions of interest (**ROI**) and performed Pearson correlation analysis on the average fMRI time trace within each ROI to calculate functional connectivity (**FC**) of each ROI permutation. Using this methodology, we commented on the functional changes introduced by comorbid SUDs as opposed to OUD alone and monitored the response of each combination of SUDs to NTX. We hypothesized that NTX would mitigate network changes due to comorbid AUD and CanUD but not CocUD.

## 2 Methods

### 2.1 Participants

Two datasets were used in the following analyses (imaging parameters for each dataset described below). For both datasets, detoxified OUD patients were recruited from the greater Philadelphia region between 2012 and 2014 and were offered up to three monthly extended release NTX injections. The DSM‐IV‐TR diagnoses of opioid use disorder (heroin or pill opioids) and comorbid SUDs were established with history and physical exam and the clinical interview. Inclusion criteria were ages between 18 and 60 years; a DSM‐IV‐TR diagnosis of opioid dependence established with history and physical exam, clinical interview self‐report and medical records documenting daily opioid use for more than 2 weeks in the past 3 months; evidence of detoxification from opioids before extended release NTX injections as established by urine drug screen (UDS) (Redwood Toxicology Laboratory) and a negative naloxone challenge test; and good physical health ascertained by history and physical examination, blood chemistry and urinalysis. Exclusion criteria were current use of medications that could confound blood oxygen level‐dependent brain response, such as antidopaminergic agents, anticonvulsants and *β*‐blockers; current psychosis, dementia, intellectual disability or lifetime history of schizophrenia; clinically significant cardiovascular, hematologic, pulmonary, hepatic, renal, metabolic, gastrointestinal, neurologic or endocrine abnormalities; pregnancy or breastfeeding; history of clinically significant head trauma; contraindications for extended release NTX, such as medical conditions requiring opioid analgesics such as chronic pain disorder, planned surgery, obesity, elevated liver enzymes > 3 times the upper limit of normal or failure to complete opioid detoxification; contraindications for MRI, such as indwelling magnetically active foreign bodies or fear of enclosed spaces [29, 35]. Both datasets were collected following protocols approved by the university's Institutional Review Board, and all subjects signed voluntary consent to treatment and imaging sessions.

A total of 69 subjects participated in the baseline imaging session (**pre‐NTX**). Data quality screening removed subjects due to motion artefact (*N* = 4; see criteria below), incomplete data (*N* = 3) or FC matrices that were statistical outliers (*N* = 2). A total of 60 subjects participated in the second imaging session (**on‐NTX**). Data quality screening removed subjects due to motion artefact (*N* = 1), incomplete data (*N* = 3) or FC matrices that were statistical outliers (*N* = 1). As the purpose of this analysis was to differentiate FC changes with respect to comorbid SUDs on top of OUD, subjects with years of opioid use that were statistical outliers were excluded from the following analysis (*N* = 5, Figure S1A) at both time points. Subjects included within each group and at each time point are listed in Table 1. To approximate recency of use and additional substance use in the absence of a SUD diagnosis, we tabulated the UDS markers for each subject (Table 1), which were collected prior to each scan. General demographic information is tabulated for each group pre‐NTX and on‐NTX (Tables 2, 3) and was not significantly different across groups by 1‐way ANOVA.

**TABLE 1:** List of subjects included at each time point with UDS (cocaine, THC and opioids) data.

Subjects	Pre‐NTX	On‐NTX	Cocaine	THC	Opioid
AUD+OUD	Subject01	Subject01			
	Subject02	Subject02			
	Subject03				+
	Subject04	Subject04			
	Subject05	Subject05			
		Subject06			
	Subject07	Subject07	+ (on‐NTX only)		
CocUD+OUD	Subject08	Subject08	+	+ (pre‐NTX only)	
	Subject09	Subject09			
	Subject10	Subject10			
	Subject11	Subject11			
		Subject12			
	Subject13				
	Subject14	Subject14			
	Subject15	Subject15			
CanUD+OUD	Subject16	Subject16		+	
	Subject17	Subject17		+	
		Subject18	+	+	
	Subject19	Subject19		+	
	Subject20	Subject20		+	
	Subject21	Subject21		+	
	Subject22	Subject22		+	
	Subject23			+	+
	Subject24	Subject24		+ (on‐NTX only)	
	Subject25	Subject25			
	Subject26	Subject26			
OUD‐only	Subject27	Subject27			+ (pre‐NTX only)
	Subject28			+	+
	Subject29				
	Subject30	Subject30		+	
	Subject31	Subject31		+	
	Subject32	Subject32			
	Subject33			+	
	Subject34 *(No UDS)	Subject34			
		Subject35		+	
	Subject36	Subject36		+	
	Subject37	Subject37			
	Subject38	Subject38			
	Subject39	Subject39			
	Subject40	Subject40			
		Subject41			
	Subject42	Subject42	+		
	Subject43	Subject43			
	Subject44				+
	Subject45			+	
	Subject46	Subject46			
	Subject47	Subject47			
	Subject48				+
	Subject49	Subject49			
	Subject50				
	Subject51	Subject51		+	
	Subject52	Subject52	+ (pre‐NTX only)	+	
	Subject53	Subject53	+ (on‐NTX only)	+ (on‐NTX only)	+ (on‐NTX only)

*Note:* For subjects included at both time points, a positive indicates they were positive for the indicated substance at both time points. No positive sign indicates a negative UDS result, which was also consistent at both time points for applicable subjects. Results specific to a time point are as indicated in parenthesis.

**TABLE 2:** Demographics across groups pre‐NTX.

Group pre‐NTX	Age	% Male	% White	% Hispanic	Years of use	Days from detox	ASI‐d	ASI‐p	CPD
OUD+CanUD	28 (9.1)	70	90	10	4.2 (3.4)	11.5 (11.4)	0.36 (0.10)	0.25 (0.32)	12.3 (9.8)
OUD+AUD	31.2 (12.6)	50	60	0	4.5 (3.1)	24.8 (34.8)	0.24 (0.07)	0.11 (0.14)	8.5 (5.2)
OUD+CocUD	26.1 (2.9)	50	70	0	5.9 (2.3)	28.4 (16.6)	0.24 (0.11)	0.26 (0.25)	14 (7.4)
OUD‐only	28.5 (6.5)	72	88	12	4.3 (2.9)	15.8 (22.5)	0.30 (0.10)	0.28 (0.25)	14.3 (8.9)
P (1‐way ANOVA)	0.7	0.95	0.36	0.63	0.09	0.58	0.67	0.67	0.5

*Note:* Values are listed as mean (standard deviation). Years of use indicate years of opioid use, ASI‐d indicates the ASI metric specific to drug use, ASI‐p indicates the ASI metric specific to psychiatric comorbidities, CPD indicates cigarettes per day. None of the demographics presented varied significantly across groups by 1‐way ANOVA.

**TABLE 3:** Demographics across groups on‐NTX.

Group on‐NTX	Age	% Male	% White	% Hispanic	Years of use	Days from detox	ASI‐d	ASI‐p	CPD
OUD+CanUD	28.2 (9.0)	70	90	20	4.4 (3.3)	23 (11.6)	0.35 (0.09)	0.23 (0.33)	13.3 (11.1)
OUD+AUD	29.3 (13.5)	40	60	0	4.8 (3.1)	37.8 (34.2)	0.22 (0.07)	0.13 (0.15)	7.5 (5.2)
OUD+CocUD	26.9 (2.9)	50	70	0	6.4 (3.5)	39.6 (19.6)	0.26 (0.12)	0.18 (0.23)	13.2 (6.9)
OUD‐only	28.2 (6.3)	70	85	0	4.6 (2.9)	30.9 (14.4)	0.29 (0.11)	0.27 (0.24)	11.9 (7.2)
P (1‐way ANOVA)	0.96	1	0.23	0.54	0.18	0.26	0.58	0.07	0.52

*Note:* Values are listed as mean (standard deviation). Years of use indicate years of opioid use, ASI‐d indicates the ASI metric specific to drug use, ASI‐p indicates the ASI metric specific to psychiatric comorbidities, CPD indicates cigarettes per day. None of the demographics presented varied significantly across groups by 1‐way ANOVA.

The same experimental timeline was used for both datasets. Initially, subjects underwent outpatient detoxification from illicit opioid use prior to the first MRI scan (pre‐NTX). Following the baseline scan, subjects received the first intramuscular NTX injection (380 mg) within the first week (*N* = 44), within the next 2–6 weeks (*N* = 4) or dropped out of the study (*N* = 7).

For the purpose of this study, subjects were categorized as OUD‐only (*N* = 25 pre‐NTX, *N* = 20 on‐NTX), CanUD+OUD (*N* = 10), AUD + OUD (*N* = 6) or CocUD+OUD (*N* = 7) (Figure S1B) at each time point. Subjects who met criteria for more than two SUDs (e.g., OUD with comorbid AUD and CanUD) were not used in the following analysis. The number of subjects included within each comparison is listed above the group‐wise averaged FC matrix in each figure.

### 2.2 Drug Use Severity

The Addiction Severity Index‐5th edition (**ASI**) [36] cumulative score served as a marker for drug use severity in each subject. Components of the ASI metric represent factors such as medical status, employment and support, drug use, alcohol use, legal status, family/social status and psychiatric status. For drug use, a cumulative score is given to rate the severity across all substances of abuse (except alcohol). In the following analysis, we reference the respective ASI metrics corresponding to substance use and psychiatric status (ASI‐d or ASI‐p, respectively, Tables 2, 3) and show by 1‐way ANOVA that these components were not significantly different across groups.

### 2.3 Image Acquisition and Preprocessing

The imaging data were collected on a Siemens Trio 3T scanner (Siemens AG, Erlangen, Germany). The first rs‐fMRI dataset (previously described [29]) was collected using a whole‐brain, single‐shot gradient‐echo echo‐planar sequence with repetition time (TR)/echo time (TE) = 2000/30 ms, field of view (FOV) = 220 × 220 mm^2^, matrix = 64 × 64, slice thickness/gap = 4.5/0 mm, 32 slices, with effective voxel resolution of 3.4 × 3.4 × 4.5 mm^3^, flip angle (FA) = 90°. The second rs‐fMRI dataset was collected with the same settings except for TR/TE = 3000/32 ms, FOV = 192 × 192 mm^2^, matrix = 64 × 64, slice thickness/gap = 3/0 mm, 46 slices, with effective voxel resolution of 3 × 3 × 3 mm^3^, FA = 90°. Resting state scans were recorded for 5 min for Dataset 1 and 6.2 min for Dataset 2. Prior work has demonstrated that sampling error is minimized after 300 s of scan acquisition [37]. For structural imaging (previously described [35]), the magnetization‐prepared rapid acquisition gradient‐echo sequence acquired high‐resolution T1‐weighted whole‐brain images with TR/TE = 1510/3.71 ms, FOV = 256 × 192 mm^2^, matrix = 256 × 192, slice thickness/gap = 1/0 mm, 160 slices, with effective voxel resolution of 1 × 1 × 1 mm^3^, FA = 9°.

The rs‐fMRI data were preprocessed in MATLAB using a pipeline adapted from Ciric and colleagues [38]. This consisted of removing the first five volumes, estimation of the 24 motion parameters (including the six raw motion parameters, six framewise displacement [**FD**] parameters, the square of the raw motion parameters and the square of the FD parameters), identification of FD time points (> 0.5 mm), removal of subjects with absolute maximum displacement from the first frame > 3 mm, identification of slice time correction, motion correction, coregistration and segmentation of the structural images, skull stripping, computation of DVARS and identification of DVARS outliers using the procedure described in Afyouni and Nichols [39], despiking using AFNI's 3dDespike, removal of polynomial trends (order = 3), extraction of nuisance signals from the voxels located within the top 10% of the deepest tissue of the white matter and the cerebrospinal fluid, interpolation of FD and DVARS outlier time points using Lomb–Scargle periodogram, bandpass filtering at 0.01–0.1 Hz of the images and covariates (i.e., 24 motion parameters and two nuisance signals), regressing out the filtered covariates, spatial smoothing using a Gaussian kernel with a full width at half maximum of 8 mm and spatial normalization to the Montreal Neurological Institute space.

### 2.4 Functional Connectivity Analysis

The Power‐264 atlas [40] was used to define a set of nonsymmetric 264 5‐mm radius evenly distributed spherical ROIs and the average time trace within these ROIs was extracted. These ROIs were distributed among 13 networks: ‘association’ networks (default mode, frontoparietal, medial parietal, ventral attention, dorsal attention, cingulo‐opercular and salience); ‘sensorimotor’ networks (hand sensory motor, mouth sensory motor, visual and auditory); and ‘other’ networks (subcortical and cerebellar). A Fisher Z‐transformed Pearson's correlation coefficient served as a measure of FC among the permuted ROI time traces.

### 2.5 Statistical Analyses

Intergroup statistical comparison of FC matrices was performed at each time point between the two groups described in each figure (either OUD‐only vs. CanUD+OUD, OUD‐only vs. AUD + OUD or OUD‐only vs. CocUD + OUD) via two‐sample *t*‐test. To correct the statistical matrix for multiple comparisons, we implemented a threshold‐free network‐based statistical (**TFNBS**) method, as described elsewhere [41]. Briefly, the statistical matrix gets iteratively thresholded (h) from zero to the maximum statistical value (thresholding set at 101 steps, dh). At each threshold, connected components are identified and each matrix index is replaced with the size of the component that index sits in ($e\left(h\right)$). Then the values within each index are summed across threshold steps to create the TFNBS matrix, whereas:

$$\text{TFNBS}\left(i,j\right)=\int_{h_{0}}^{h\left(i,j\right)}e{\left(h\right)}^{E}h^{H}\mathrm{dh}$$

where i and j index the statistical matrix, $e\left(h\right)$ is the component size at threshold h, and E and H are the extension and height parameters as defined by Baggio et al. (E=0.5, H=2.25) [41]. Compared to a null TFNBS matrix (creating through group shuffling, *n* = 3000 permutations), only the observed TFNBS values for *p* < 0.05 are displayed in the end result.

In order to compare the overall change in correlation values with NTX, we took the difference in FC between each SUD+OUD subject and the average OUD‐only group pre‐ and on‐NTX. We then took the summation of correlation differences across each network. Only ROIs that reached statistical significance via TFNBS pre‐NTX were included to avoid difference calculations being heavily weighted by nonsignificant ROIs. Statistical significance was determined by two‐sample *t*‐test comparing pre‐ and on‐NTX values by network, and the threshold for significance was set at *p* = 0.05/13 (*N* networks = 13, i.e., Bonferroni correction) [42]. To visualize the most affected brain regions, we took the top 5% individual ROIs with the largest differences in TFNBS values pre‐ vs. on‐NTX and mapped these on a brain atlas, colour‐coded by network. The edges connecting these ROIs represent the difference in connectivity values pre‐ and on‐NTX with larger bars indicating larger differences [43].

Intragroup analyses were calculated by averaging FC values within the most sampled region, which also displayed the highest degree of hyperconnectivity in the CanUD+OUD group, the default mode network (**DMN**). This measure was used in regression analyses with multiple demographic factors and tested for differences in mean values pre‐ and on‐NTX by two‐sample *t*‐test. Values were plotted pre‐NTX and on‐NTX with dotted lines connecting repeated measurements. For intergroup analysis, an ANOVA was used to test for significance of group interaction (comorbid SUD+OUD vs. OUD‐only) by time.

In addition to single variable regression with DMN FC for exploratory analyses, we performed multivariate regression with average Pearson FC within DMN as the dependent variable and group (either OUD‐only or comorbid SUD+OUD), age and sex as independent predictors.

*p*‐values for all statistical testing are organized by figure in Tables 4, 5 in supplement.

**TABLE 4:** *p*‐values from main Figure 2.

Figure 2 two‐sample *t*‐test by network.													
	Default	ventatn	doratn	salience	fronpar	medpar	subcort	cblm	cingop	SMHand	SMMouth	aud	vis
CanUD+OUD	0*	0*	0.0013*	0.2096	0.0073	0.0006*	0.0009*	0.1372	0.6617	0.0031*	0.021	0.0023*	0.0002*
AUD+OUD	0.0035*	0.0077	0.0109	0.0021*	0.0006*	0.0369	0.0011*	0.0013*	0.0008*	0.008	0.0002*	0.0031*	0.0371
CocUD+OUD	0.8224	0.4598	0.1175	0.2252	0.9259	0.2373	0.2866	0.554	0.956	0.1139	0.1653	0.3177	0.2694

* *p* < 0.05.

**TABLE 5:** *p*‐values from main Figure 3 by two‐sample *t*‐test or ANOVA.

Figure 3 two‐sample *t*‐test	
Pre‐ vs. on‐NTX	DMN
CanUD+OUD	0.027*
AUD+OUD	0.64
CocUD+OUD	0.45
OUD‐only	0.73
Figure 3 ANOVA	
Group × time	DMN
CanUD+OUD	0.04*
AUD+OUD	0.57
CocUD+OUD	0.65

* *p* < 0.05.

## 3 Results

### 3.1 Pre‐NTX, CanUD+OUD, AUD + OUD and CocUD+OUD Are All Associated With Unique FC Alterations Compared to OUD‐Only. After NTX, These Alterations Are Diminished in CanUD+OUD and AUD + OUD, but Not in CocUD+OUD

**FIGURE 1:**
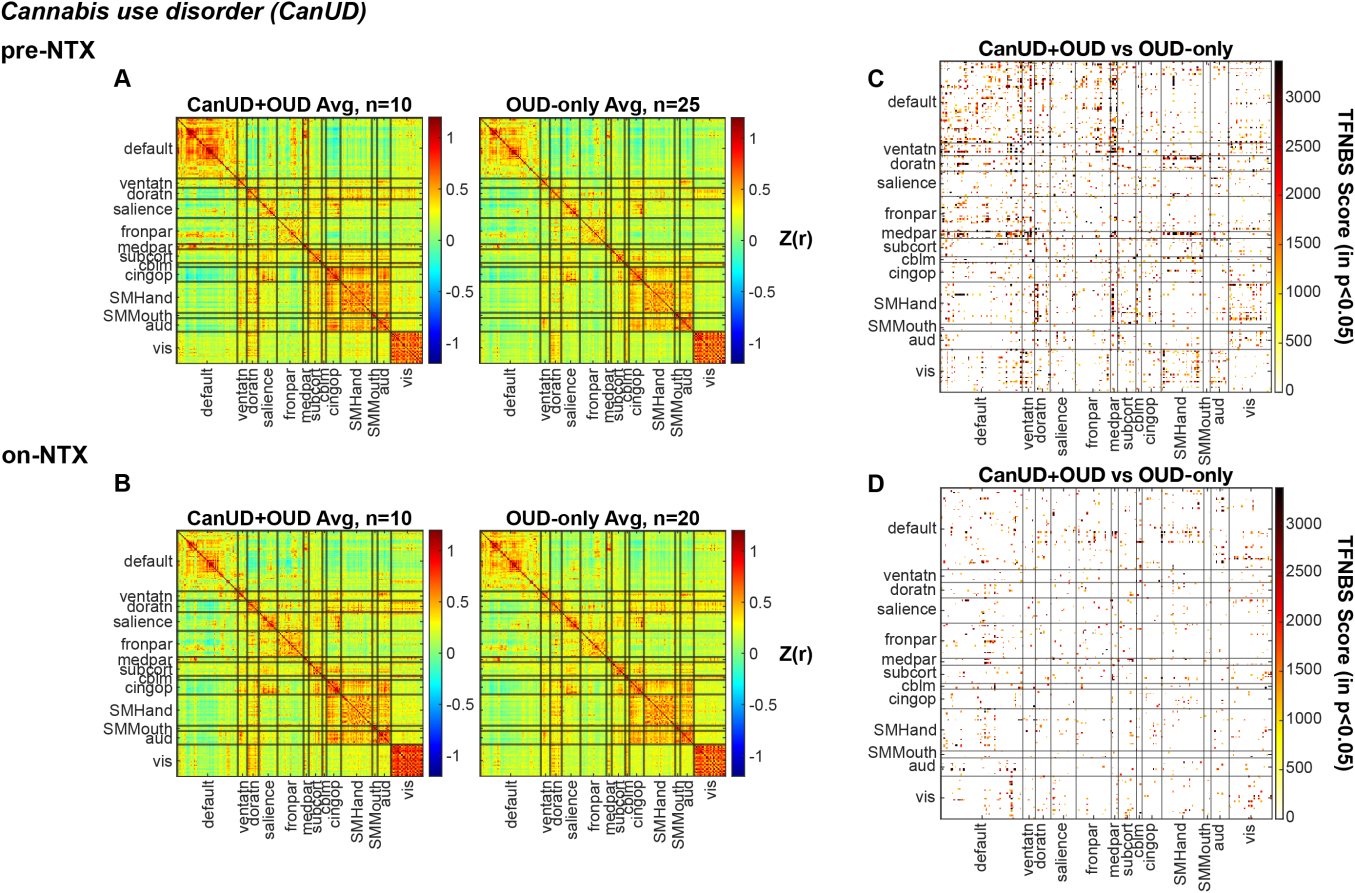
Functional connectivity is altered at baseline in CanUD+OUD compared to OUD‐only, but these differences decrease with NTX. Pearson correlation coefficients (*r*) representing the functional connection strength between two ROI's within networks specified on the *x*‐ and *y*‐axis at (A) baseline and (B) after receiving NTX. Matrices are organized to display FC values for (left to right) CanUD+OUD and OUD‐only. Matrices displaying the TFNBS scores of CanUD+OUD vs. OUD‐only comparisons with *p* < 0.05 by two‐sample *t*‐test at (C) baseline and (D) after receiving NTX.

Following detoxification and prior to administering NTX, *N* = 10 subjects with CanUD+OUD underwent resting state imaging, along with *N* = 25 subjects with OUD‐only. By determining the Pearson correlation coefficient between all combinations of ROIs defined by the Power atlas, the FC of inter‐ and intrabrain networks was calculated (Figure 1A). At roughly 2 weeks post NTX injection, N = 10 subjects with CanUD+OUD underwent resting state imaging, along with *N* = 20 controls with OUD‐only and the same FC calculation was done on this data (Figure 1B). To statistically compare the two groups at each time point, a two‐sample *t*‐test was performed on each ROI x ROI FC value. To correct for multiple comparisons, a TFNBS approach was used to place more statistical weight on heavily interconnected graph components, with greater differences appreciated between groups pre‐NTX (Figure 1C) than on‐NTX (Figure 1D).

Using the same experimental timeline and computational model as in Figure 1, the same comparisons were performed using *N* = 6 subjects with AUD + OUD (compared to the same *N* = 25 or N = 20 subjects with OUD‐only, pre‐NTX or on‐NTX, respectively, Figures S2A,B). The same TFNBS approach was used to statistically compare the two groups at each time point, and similarly to CanUD + OUD in Figure 1, greater differences in FC were found between groups pre‐NTX (Figure S2C) than on‐NTX (Figure S2D). This same analysis was repeated for *N* = 7 subjects with CocUD + OUD (Figures S3A,B) and yielded consistent statistical differences between groups at the two imaging time points (Figures S3C,D).

### 3.2 Parietal, Attention, Subcortical and Sensory Network Differences Compared to OUD‐Only Are Significantly Reduced With NTX in CanUD+OUD Whereas Parietal, Subcortical, Sensory and Cerebellar Network Differences Are Significantly Reduced in AUD + OUD. Networks Remain Relatively Unchanged With NTX in CocUD+OUD

**FIGURE 2:**
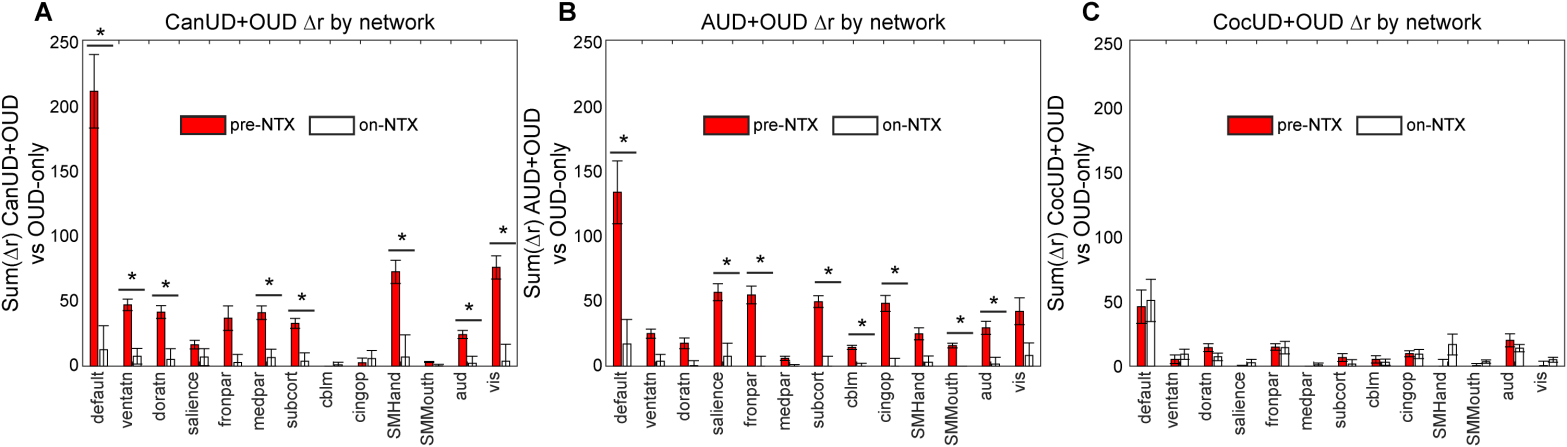
NTX has a normalizing effect on most networks in CanUD+OUD and AUD+OUD, but hardly any effect in CocUD+OUD (all compared to OUD‐only). The summation of differences in Pearson correlation values between (A) CanUD+OUD, (B) AUD + OUD and (C) CocUD+OUD compared to OUD‐only at both time points. Statistical significance is determined between values pre‐NTX and on‐NTX by two‐sample *t*‐test. Significance is determined by alpha = 0.05/13 (number of networks).

There was a striking difference in TFNBS scores pre‐ and on‐NTX in CanUD+OUD and AUD + OUD compared to OUD‐only (Figure 1C,D and Figure S2C,D), which was nicely illustrated by taking the network‐wise sum of all TFNBS values at each time point (Figure S4A–C). For CanUD+OUD and AUD + OUD, the TFNBS scores between pre‐NTX and on‐NTX decreased by more than half in most networks. This finding was not as obviously present in the analysis with CocUD+OUD (Figure S4C). Here, the total sum of TFNBS remained roughly unchanged between pre‐NTX and on‐NTX (however there was some redistribution of which networks demonstrated more/less FC changes compared to OUD‐only at the two time points). Indeed, in CanUD+OUD and AUD + OUD, the difference between these SUD + OUD FC matrices and OUD‐only FC matrices pre‐NTX were mostly due to hyperconnectivity in the SUD + OUD condition (increased positive correlations in Figure S5, top row). The differences between CanUD+OUD or AUD + OUD and OUD‐only on‐NTX were reduced, whereas multiple differences were still noted in CocUD+OUD (Figure S5, bottom row). To quantify this, we took the difference in FC between each SUD + OUD subject and the average OUD‐only group pre‐ and on‐NTX and calculated the sum of differences by network (Figure 2). Eight of the 13 networks evaluated had significant decreases in connectivity between time points in the CanUD+OUD and AUD + OUD groups (Figures 2A,B). No such decrease was appreciated in any network in the CocUD+OUD group (Figure 2C). Spatially mapping the ROIs of greatest FC difference illustrated that the majority of most affected ROIs rest within default mode network, specifically the regions of DMN in the bilateral frontal lobes (Figure S6).

### 3.3 Only in CanUD+OUD Did DMN FC Correlate With ASI‐d. Also in CanUD+OUD, FC Alterations Within DMN Were Larger in Younger Brains Compared to in OUD‐Only

**FIGURE 3:**
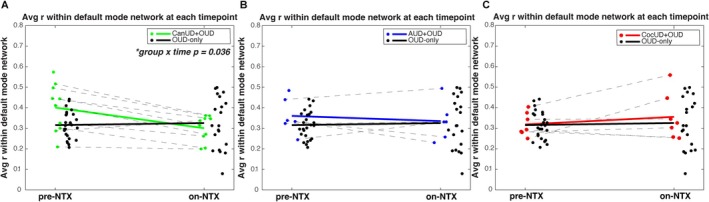
Inter‐ and intragroup comparisons before and after NTX within DMN. Average individual Pearson correlation values averaged across the DMN per subject at each time point. Dots represent each subject with solid lines representing average values in (A) CanUD+OUD, (B) AUD + OUD and (C) CocUD+OUD. Dashes determine repeat measures. Significance determined by ANOVA, with only CanUD+OUD having a significant group × time effect. Intragroup significance determined by two‐sample *t*‐test with only CanUD+OUD group reaching significance (Table 5).

Notably, the DMN has the highest density of ROIs per the Power atlas, which partially explains the large TFNBS score observed in this network (Figure S4A). However, in CanUD+OUD, using both the TFNBS approach (Figure S4A) and the correlation difference approach (the latter not dependent on ROI number, Figure 2A), the DMN had the greatest change in FC strength on‐NTX. We used this network to illustrate each individual subject trajectory from pre‐NTX to on‐NTX. We calculated the average FC within DMN for each group and plotted individual subjects pre‐NTX and on‐NTX (Figure 3). When comparing inter‐ and intragroup changes on‐NTX, there was a significant decrease in FC in DMN in the CanUD+OUD groups (calculated by ANOVA, significant group × time interaction, Figure 3A). Also, only in the CanUD+OUD group, there was a significant decrease in DMN FC on‐NTX determined by two‐sample *t*‐test (*p* = 0.027, Table 5). Neither ANOVA nor two‐sample *t*‐tests yielded significant results in AUD + OUD or CocUD+OUD (Figure 3B,C, Table 5). Further, the relationship between average FC in DMN was inversely related to age in the CanUD+OUD group and also in the OUD‐only group (Figures S7B,C). The inverse relationship was stronger in the CanUD+OUD group compared to the OUD‐only group (*R* ^2^ = 0.64 vs. *R* ^2^ = 0.12). Other regressions between DMN FC and ASI‐d, and DMN FC and years of opioid use were only significant for CanUD+OUD (Table 6). For CanUD+OUD and AUD + OUD, group, age and sex were significant predictors of DMN FC pre‐NTX, whereas none were significant predictors on‐NTX (Table 7).

**TABLE 6:** Single variable regression between average Pearson *r* within DMN in each condition pre‐NTX with either (top) days from detox, (middle) ASI‐d or (bottom) years of opioid use.

Days from detox		CanUD+OUD	AUD + OUD	CocUD+OuD	OUD‐only
	*R* ^2^	0.12	0.14	0.04	0.04
	*p*	0.94	0.57	0.31	0.94
ASI‐d		CanUD+OUD	AUD+OUD	CocUD+OuD	OUD‐only
	*R* ^2^	0.42	−0.33	−0.31	0.08
	*p*	0.03*	0.94	0.11	0.09
Years opioid use		CanUD+OUD	AUD+OUD	CocUD+OuD	OUD‐only
	*R* ^2^	−0.47	−0.15	−0.19	0.04
	*p*	0.02*	0.24	0.82	0.82

* *p* < 0.05.

**TABLE 7:** Multivariate regression using comorbid SUD group, age and sex, compared to OUD‐only group.

DMN ~ group + age + sex	CanUD+OUD		AUD + OUD		CocUD+OUD	
	Pre‐NTX	On‐NTX	Pre‐NTX	On‐NTX	Pre‐NTX	On‐NTX
Group	0.002*	0.56	0.03*	0.86	0.74	0.58
Age	6.1 × 10–5*	0.59	0.007*	0.9	0.013*	0.78
Sex	0.034*	0.4	0.021*	0.64	0.008*	0.36
*R* ^2^	0.5	−0.06	0.33	−0.12	0.27	−0.07
Model *p*	1.6 × 10–5*	0.7	0.003*	0.96	0.007*	0.73

*Note:* Average Pearson *r* within DMN predicted by group (either comorbid SUD listed or OUD‐only), age and sex. *p*‐values for each variable are displayed as well as *R* ^2^ and *p*‐value for the model.

* *p* < 0.05.

## 4 Discussion

This study represents secondary analyses to investigate the differential effect of NTX on functional network connectivity in OUD subjects with comorbid CanUD, AUD or CocUD. Pearson FC analysis was performed at baseline and roughly 2 weeks after a therapeutic dose of long‐acting injectable NTX. A graph‐theory–based statistical approach yielded large and significant differences in FC between each SUD+OUD condition and OUD‐only pre‐NTX that mostly resolved on‐NTX in CanUD+OUD and AUD+OUD, but not in CocUD+OUD. Although sample sizes were limited in the present analysis, we present concordant findings via intergroup (SUD + OUD vs. OUD‐only) and intragroup (pre‐NTX vs. on‐NTX) analysis in CanUD+OUD (i.e., decrease in FC on‐NTX for subjects with CanUD+OUD when compared to OUD‐only in Figures 1, 2 or when compared within group in Figure 3 and Table 5). Although NTX is an established treatment for OUD and AUD, no medication treatment has been established for CanUD. Specifically, in CanUD+OUD, the default mode network (**DMN**) was the most affected at baseline with bigger FC alterations present in younger subjects.

Functional connectivity is a non‐invasive surrogate measure of coordinated neural activity [44] and has been robustly shown to be sensitive to underlying disease processes in humans [32, 34] and animal models [45, 46]. Both degradation and enhancement of FC strength among networks can be pathological. In fact, in neuroimaging studies focused on cognitive impairment and unhealthy aging, main findings have dictated a period of functional hyperconnectivity preceding the late‐stage hypoconnectivity that is usually found in steep cognitive decline/dementia [47]. In SUDs, recent neuroimaging studies in OUD and AUD have described a degradation of functional network segregation, an expression that quantifies the overall loss of variance in FC values (e.g., globally hyper‐ or hypoconnected brains) [28, 29]. Fortunately, despite changes in brain architecture, brains have a remarkable ability to rewire and regain function (i.e., plasticity). In otherwise healthy individuals this can occur in as little as 48 h [33]. Although the presented time‐course of this study is rather short (~2 weeks), the inclusion/exclusion criteria resulted in a sample of relatively healthy individuals, outside of their SUD(s), making the 2‐week time point a reasonable interval to expect plasticity to occur. In that time, the differences between CanUD+OUD or AUD + OUD and OUD‐only significantly decreased from pre‐NTX to on‐NTX (Figure 2A,B). FC measures were more uniform in comorbid CocUD at both time points (Figure 2C).

Since the findings related to OUD‐only have been previously described [48, 49], the goal of the present analysis was to delineate functional alterations due to comorbid SUDs, while controlling for opioid use. To this end, all comorbid SUD comparisons were against OUD subjects without any other comorbid SUDs, and five subjects were removed based on heavier opioid use compared to the rest of the group (Figure S1A). As expected, years of opioid use moderately correlated with age of subject (*r* = 0.47), but neither demographic (nor any demographic reported) was significantly different across groups (Tables 2, 3). There was a slight inverse correlation between age and ASI‐d (*r* = −0.22), and no correlation with ASI‐p (*r* = −0.03), which mildly suggests younger subjects have more complications due to drug use that are not psychiatric in nature. It is possible that a subject may use a substance but not meet criteria for a SUD (therefore contributing to the heterogeneity of results); however, UDS data suggested minimal cross‐substance use outside of the SUD diagnoses collected (Table 1). Further, according to the average time from detox to each scan (10 or more days) as represented in Tables 2, 3, withdrawal from heroin or pill opioids (i.e., short acting opioids) was unlikely to be occurring at the time of each scan, and there was no significant relationship between days since detox and DMN FC within any group (Table 6). Subjects were only consented to be treated for OUD and not required to abstain from other substances. Though nearly all subjects with repeated measures had consistent UDS results (Table 1), it is still possible that withdrawal from any substance could be contributing to the present results. However, in the cases of CanUD+OUD and AUD + OUD, age and sex (in addition to group) were significant predictors of DMN FC pre‐NTX; this effect was not appreciated on‐NTX (Table 7). The regression model was unable to differentiate comorbid SUD+OUD and OUD‐only at the on‐NTX time point, which we believe is further evidence that NTX has mitigating effects on the network disturbances induced by these comorbid conditions.

Using the TFNBS statistical methods described, the largest change in FC after NTX was within default mode network in CanUD+OUD. The DMN is foundational in the brain's functional integrity and internal mentation at rest but also gets activated during episodic memory and predicting future tasks, processes that are frequently disrupted in psychiatric illness (e.g., schizophrenia and depression) [50, 51]. Interestingly, some of the greatest changes in FC affect the frontal areas of the DMN across all groups (Figure S6), which is heavily implicated in addiction and integral for impulse control processing [52]. Made up of the ventral medial prefrontal cortex, the dorsal medial prefrontal cortex and the posterior cingulate cortex, the DMN is spatially disjointed from the dorsal attention network (**DAN**). Also functionally antagonistic, the DAN is primarily indicated in visuospatial attention and therefore utilized in most task‐based behaviours. Made up of the intraparietal sulcus and frontal eye fields, the DAN primarily integrates sensory information to coordinate a motor response [53]. A third constellation of brain regions has been shown to be a functional mediator of the DMN and DAN during goal‐directed activity [54]. The frontoparietal network is made up of the dorsolateral prefrontal cortex and posterior parietal cortex and largely thought to be the control centre of salience processing and executive function [55]. The frontoparietal network has been shown to functionally interplay between the DMN and DAN, with nodes aligned with each as well as aligned with both, to complete a triangular unit that modulates an individual's ability to incorporate and respond to internal and external stimuli [56, 57]. Functional alteration within any of these three networks has been described in patients at high risk of psychosis [58], experiencing psychotic‐like episodes [59], and those without psychosis but with a history of cannabis use [60]. Further, this constellation of networks, as well as interactions with the salience network, has been shown to influence drug‐taking behaviours and be associated with negative emotions, ruminations and impaired self‐awareness [61]. This is consistent with the findings from behavioural cannabis studies in adolescents showing a decline in working memory, attention and executive functioning into adulthood [62]. It is concerning that our analysis here shows a larger baseline effect in younger individuals (Figure S7), which could suggest the need for earlier intervention in CanUD. Additionally, only CanUD+OUD had a significant relationship between DMN FC and metrics of substance use severity (ASI‐d and total years of opioid use). There was a positive relationship between FC and ASI‐d but an inverse relationship between FC and years of opioid use (Table 6). The CanUD+OUD group exhibited the highest connectivity within DMN at baseline (Figure 2A), and these values scaled with fewer years of opioid use but higher ASI‐d scores. Though we lack the necessary data here to determine whether this was due to cannabis use or more severe, short‐term opioid usage, future experiments will investigate how varying severity of cannabis versus opioid use impacts FC. Nevertheless, the results here are encouraging in that NTX mitigated these FC alterations in all the aforementioned networks in CanUD+OUD (Figure 2A).

In AUD + OUD, parietal, sensory and cerebellar were the main networks affected by NTX. Unique to AUD + OUD is the decrease in network differences with OUD‐only in cerebellar FC after NTX. In AUD, multiple studies document cerebellar dysfunction both in acute intoxication from alcohol and in chronic AUD. From in utero development, where exposure to alcohol induces multiple cerebellar deficits [63], to the acute intoxication period in adolescence/adulthood, where high blood alcohol levels start to impact functions governed by the cerebellum (e.g., coordination), to long‐term use in AUD, ethanol impacts GABA signalling in Purkinje cells as well as interneurons and granule cells that fortify the neural transmission pathways of the cerebellum [64]. Elsewhere, connectivity with cerebellar networks predicted response to brief interventions with NTX in AUD [65]. The baseline FC alterations in CocUD+OUD compared to OUD‐only were less pronounced than in CanUD+OUD and AUD + OUD, but still present (Figure S3C). In a recent study, CocUD manifested in a unique FC signature that was distinguishable from controls, involving internetwork connections in frontoparietal, default mode, dorsal attention, limbic, ventral attention, visual and somatomotor networks, which was also evidenced here (Figures S3C and S4C) [66]. Unfortunately, treatment with NTX did not mitigate any of these findings.

As mentioned above, there is no FDA‐approved treatment of CanUD. NTX, however, is approved for the treatment of both OUD and AUD. The mechanism by which NTX addresses alcohol misuse is through decreasing the perceived reward associated with drinking [21]. This decreased reward has also been reported in CanUD with NTX [25], though the effectiveness of NTX for CanUD needs further investigation. Notably, NTX has been ineffective for CocUD, [22]. Therefore, the present imaging results in AUD + OUD and CocUD+OUD are consistent with the current clinical evidence for NTX to treat either disorder suggesting these FC findings to be a useful biomarker for tracking treatment response; with AUD, NTX mitigated functional network alterations; in CocUD, it seemed to have little effect. The results in CanUD+OUD were promising, as FC alterations were significantly mitigated after a single dose, similar to the response seen in AUD + OUD. Although the ideal treatment for a SUD would involve long‐term maintenance of network activity indicated in addiction (e.g., reward, executive and memory networks), the ability for NTX to affect these networks long‐term is outside the scope of this work. Instead, we present further evidence that NTX can aide to acutely restore some of the early functional changes present in patients with OUD and comorbid CanUD.

## 5 Limitations

The present study was a secondary analysis conducted on data collected on primary OUD subjects with and without comorbid CanUD, AUD and CocUD. One limitation of the present work includes the lack of a non‐OUD group for comparisons, which will be explored in future work. Although UDS data were used to approximate recency of substance use (and therefore the effect of substance detox), metabolites detected on UDS are cleared at different rates depending on the substance so a future trial with a placebo medication group can best inform on NTX‐specific versus withdrawal effects. Additionally, sample sizes are limited within this study; however, we present two methods to statistically test our findings (intergroup and intragroup) with converging results. Although ANOVA and two‐sample *t*‐testing of the data revealed inter‐ and intragroup differences in FC with the CanUD+OUD group, these differences were not present within the AUD + OUD, CocUD+OUD or OUD‐only data (although AUD + OUD showed intergroup differences vs. OUD‐only by the TFNBS approach). This is likely due to the moderate to large change in FC seen within DMN in CanUD+OUD at baseline (Figure 1A). There were less striking findings in AUD + OUD and CocUD+OUD (Figures S2A, S3A); thus, while we present supporting results in AUD + OUD and CocUD+OUD, it is possible the presented analyses were not adequately powered by the number of subjects within these groups. Still, we note that the trend in the AUD + OUD group was a decrease in connectivity strength on‐NTX whereas the trend in CocUD+OUD was the opposite (Figure 3B,C). Altogether, we present multiple approaches to suggest a mitigating effect of NTX on brain networks in CanUD+OUD and AUD + OUD.

## 6 Conclusions

The present work explored the differences in FC between comorbid SUD+OUD groups and an OUD‐only group. At baseline, differences were uniquely distributed among the 13 networks analysed for the comorbid SUD + OUD groups when compared to OUD‐only. Although this suggests that the comorbid SUD induced these effects, we do lack comparison to a non‐OUD group and do not present data on subjects with each individual SUD. NTX is currently an FDA‐approved treatment for OUD via opioid antagonism; therefore, we sought to examine effects NTX may have on the additional comorbid SUD FC patterns, which would likely be through other downstream mechanisms. Within this framework, we present evidence that NTX altered CanUD+OUD networks in a similar way that AUD + OUD networks were affected, with CocUD+OUD networks remaining relatively unaffected. Using this biomarker (which in the case of comorbid AUD and CocUD could be reflective of current FDA approvals), we are encouraged to explore the role of NTX in the management of CanUD.

## Author Contributions

L.M.B. prepared the manuscript with help from other authors. L.M.B., C.E.W. and Z.S. conceived the research idea and performed the analyses. D.D.L. and Z.S. performed the experiments.

## Ethics Statement

All data were collected following protocols approved by the university's Institutional Review Board, and all subjects signed voluntary consent to treatment and imaging sessions.

## Conflicts of Interest

The authors declare no conflicts of interest.

## Supporting information


**Figure S1:** SUDs within imaged subjects. (A) Years of opioid use in all subjects considered for analysis. The five statistical outliers were removed from the analysis reported in this study. (B) Number of subjects with each substance use disorder (or combination of disorders) at each time point.

**Figure S2:** Functional connectivity is altered at baseline in AUD+OUD compared to OUD‐only, but these differences decrease with NTX. Pearson correlation coefficients (*r*) representing the functional connection strength between two ROI's within networks specified on the *x*‐ and *y*‐axis at (A) baseline and (B) after receiving NTX. Matrices are organized to display FC values for (left to right) AUD+OUD and OUD‐only. Matrices displaying the TFNBS scores of AUD + OUD vs. OUD‐only comparisons with *p* < 0.05 by two‐sample *t*‐test at (C) baseline and (D) after receiving NTX.

**Figure S3:** Functional connectivity is altered at baseline in CocUD+OUD compared to OUD‐only and is minimally affected by NTX. Pearson correlation coefficients (*r*) representing the functional connection strength between two ROI's within networks specified on the *x*‐ and *y*‐axis at (A) baseline and (B) after receiving NTX. Matrices are organized to display FC values for (left to right) CocUD+OUD and OUD‐only. Matrices displaying the TFNBS scores of CocUD+OUD versus OUD‐only comparisons with *p* < 0.05 by two‐sample *t*‐test at (C) baseline and (D) after receiving NTX.

**Figure S4:** FC differences between CanUD+OUD or AUD+OUD and OUD‐only decrease with NTX, but not in CocUD+OUD. The within‐network sum of TFNBS scores that result from comparing each comorbid SUD + OUD versus OUD‐only at baseline (in colour) or after NTX (black) in (A) CanUD+OUD (B) AUD+OUD and (C) CocUD+OUD.

**Figure S5:** Difference matrices between each SUD+OUD condition and OUD‐only either pre‐NTX or on‐NTX.

**Figure S6:** Connections with highest TFNBS scores predominantly involve DMN across the conditions presented. ROIs from the nonsymmetric Power atlas are plotted on a template brain. Only the top 5% individual ROIs with the largest differences in TFNBS values pre‐ versus on‐NTX for each condition are visualized for clarity. ROIs are colour coded by network. Each black bar is weighted to represent the magnitude of difference in Pearson correlation value for each connection pre‐ to on‐NTX for (A) CanUD+OUD, (B) AUD + OUD and (C) CocUD+OUD.

**Figure S7:** In default mode network, younger subjects had bigger FC alterations at baseline in those with CanUD+OUD. Linear regression between average Pearson correlation coefficient (*r*) within default mode network and subject age in (Left) CanUD+OUD and (Right) OUD‐only.

## Data Availability

The data that support the findings of this study are available from the authors upon reasonable request.
