# Supplementary figures and images for: Long‐Acting Naltrexone Restores Network Connectivity in Subjects With Comorbid Cannabis and Opioid Use Disorder
Source: Addict Biol. 2026 May 17;31(5):e70159. doi: 10.1111/adb.70159 (PMC13181156; doi:10.1111/adb.70159)

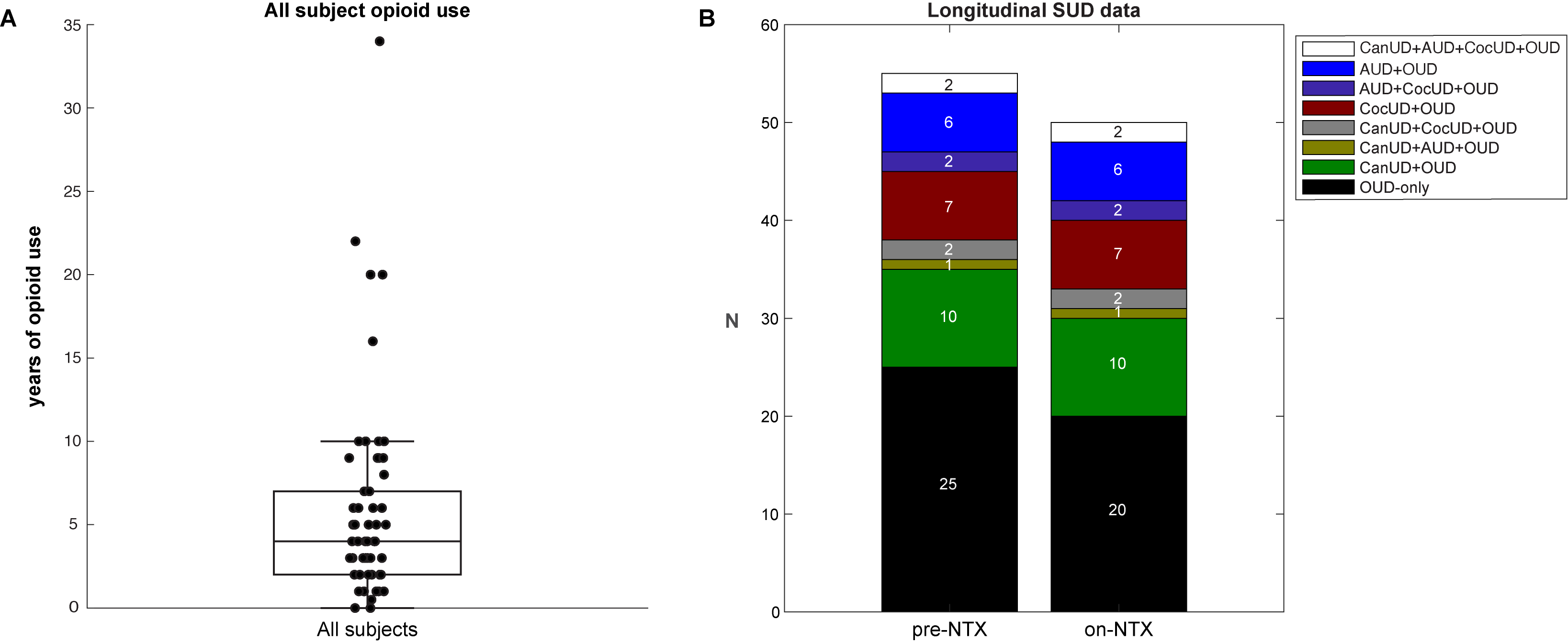

Supplement: Supplementary file 1 — Figure S1: SUDs within imaged subjects. (A) Years of opioid use in all subjects considered for analysis. The five statistical outliers were removed from the analysis reported in this study. (B) Number of subjects with each substance use disorder (or combination of disorders) at each time point. [file ADB-31-e70159-s007.BMP]

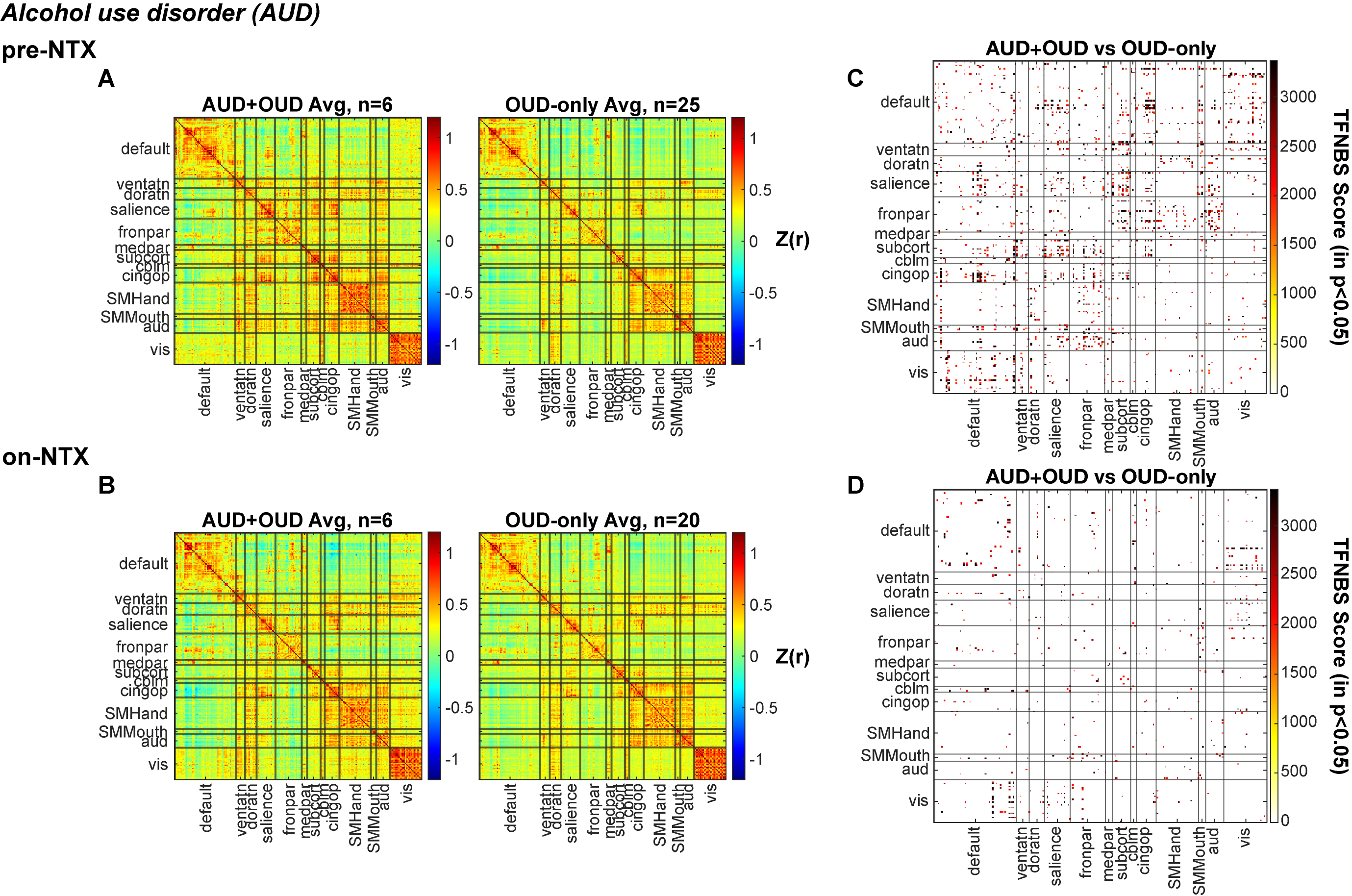

Supplement: Supplementary file 2 — Figure S2: Functional connectivity is altered at baseline in AUD+OUD compared to OUD‐only, but these differences decrease with NTX. Pearson correlation coefficients (r) representing the functional connection strength between two ROI's within networks specified on the x‐ and y‐axis at (A) baseline and (B) after receiving NTX. Matrices are organized to display FC values for (left to right) AUD+OUD and OUD‐only. Matrices displaying the TFNBS scores of AUD + OUD vs. OUD‐only comparisons with p < 0.05 by two‐sample t‐test at (C) baseline and (D) after receiving NTX. [file ADB-31-e70159-s001.BMP]

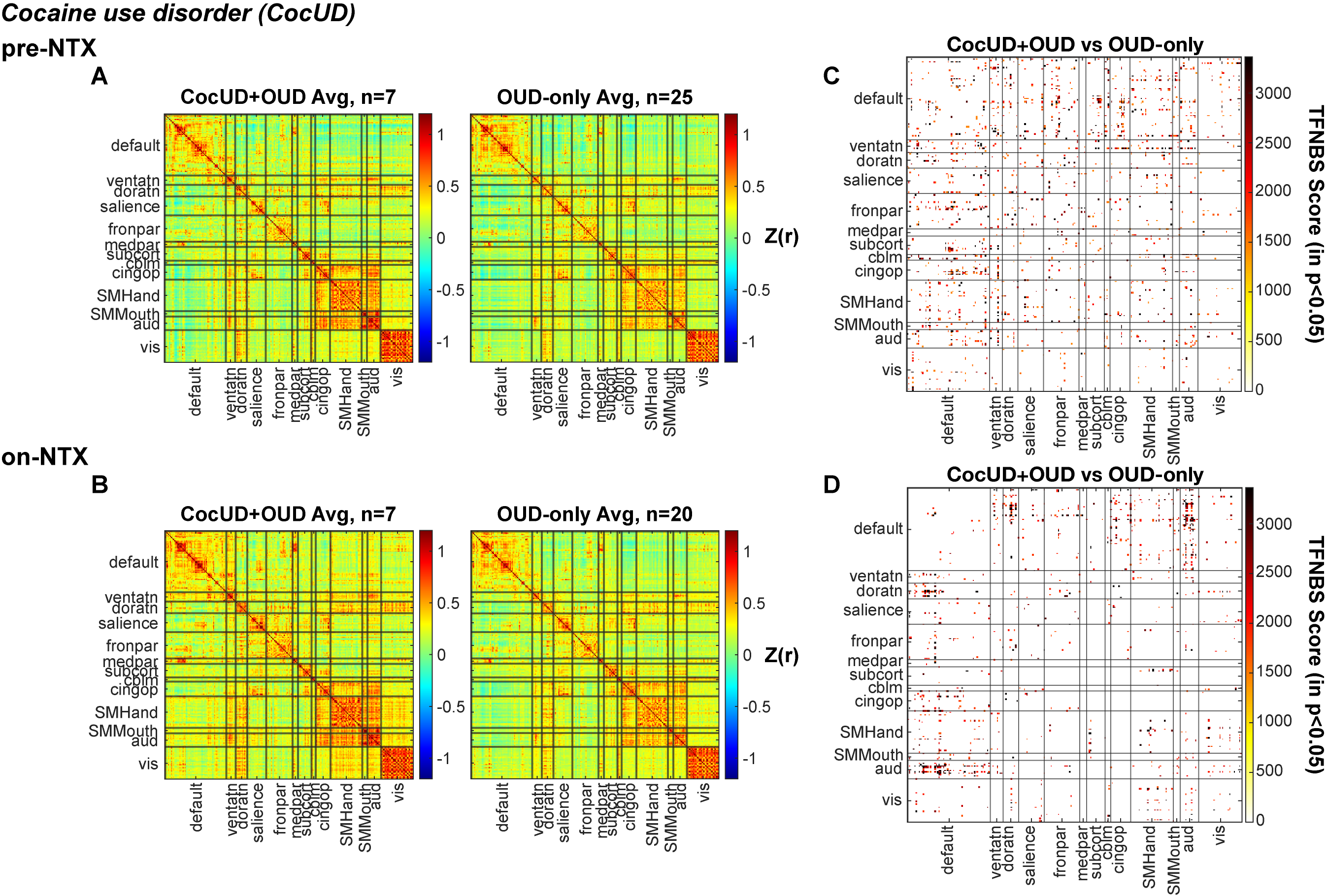

Supplement: Supplementary file 3 — Figure S3: Functional connectivity is altered at baseline in CocUD+OUD compared to OUD‐only and is minimally affected by NTX. Pearson correlation coefficients (r) representing the functional connection strength between two ROI's within networks specified on the x‐ and y‐axis at (A) baseline and (B) after receiving NTX. Matrices are organized to display FC values for (left to right) CocUD+OUD and OUD‐only. Matrices displaying the TFNBS scores of CocUD+OUD versus OUD‐only comparisons with p < 0.05 by two‐sample t‐test at (C) baseline and (D) after receiving NTX. [file ADB-31-e70159-s002.BMP]

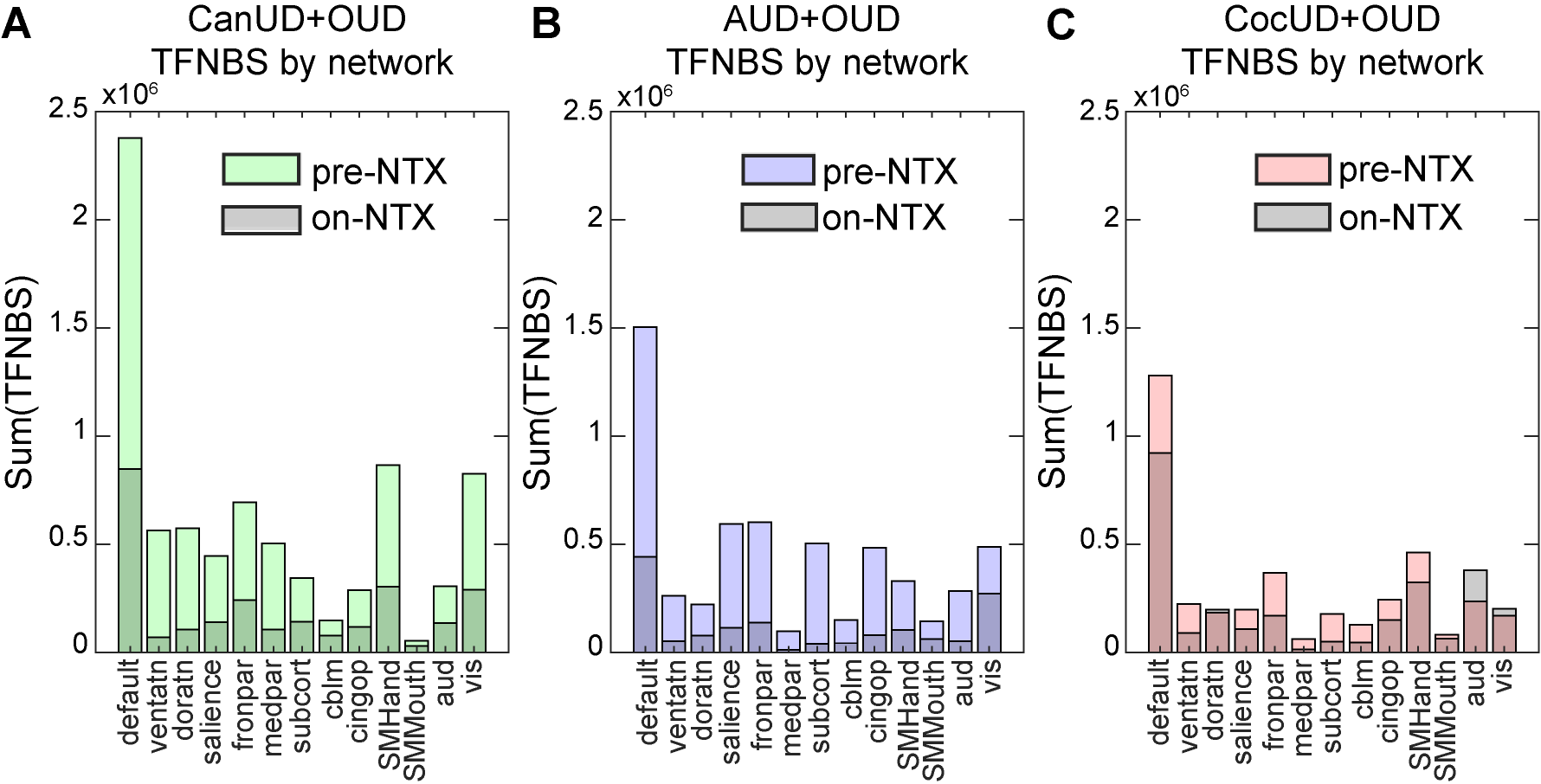

Supplement: Supplementary file 4 — Figure S4: FC differences between CanUD+OUD or AUD+OUD and OUD‐only decrease with NTX, but not in CocUD+OUD. The within‐network sum of TFNBS scores that result from comparing each comorbid SUD + OUD versus OUD‐only at baseline (in colour) or after NTX (black) in (A) CanUD+OUD (B) AUD+OUD and (C) CocUD+OUD. [file ADB-31-e70159-s006.BMP]

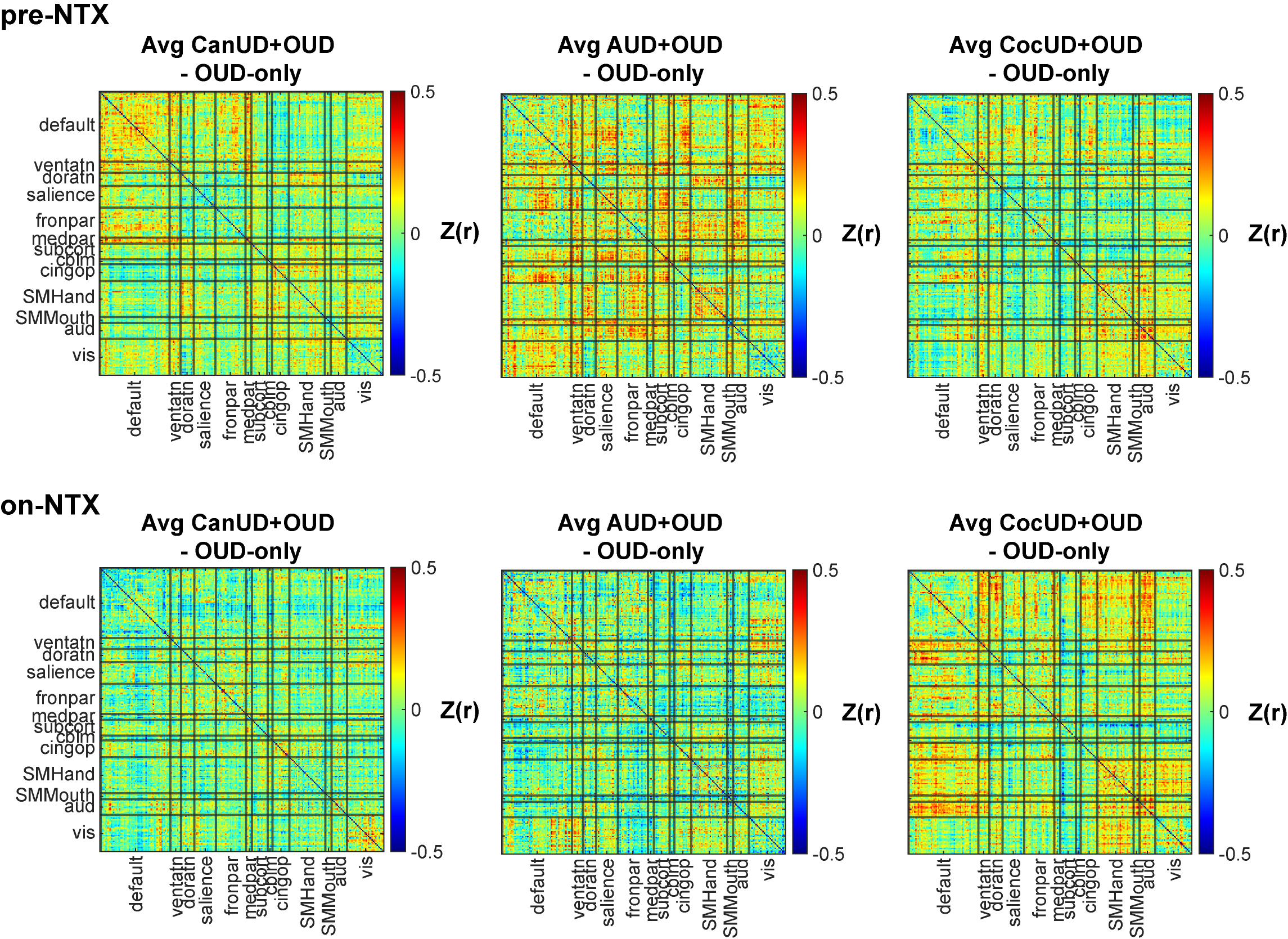

Supplement: Supplementary file 5 — Figure S5: Difference matrices between each SUD+OUD condition and OUD‐only either pre‐NTX or on‐NTX. [file ADB-31-e70159-s003.BMP]

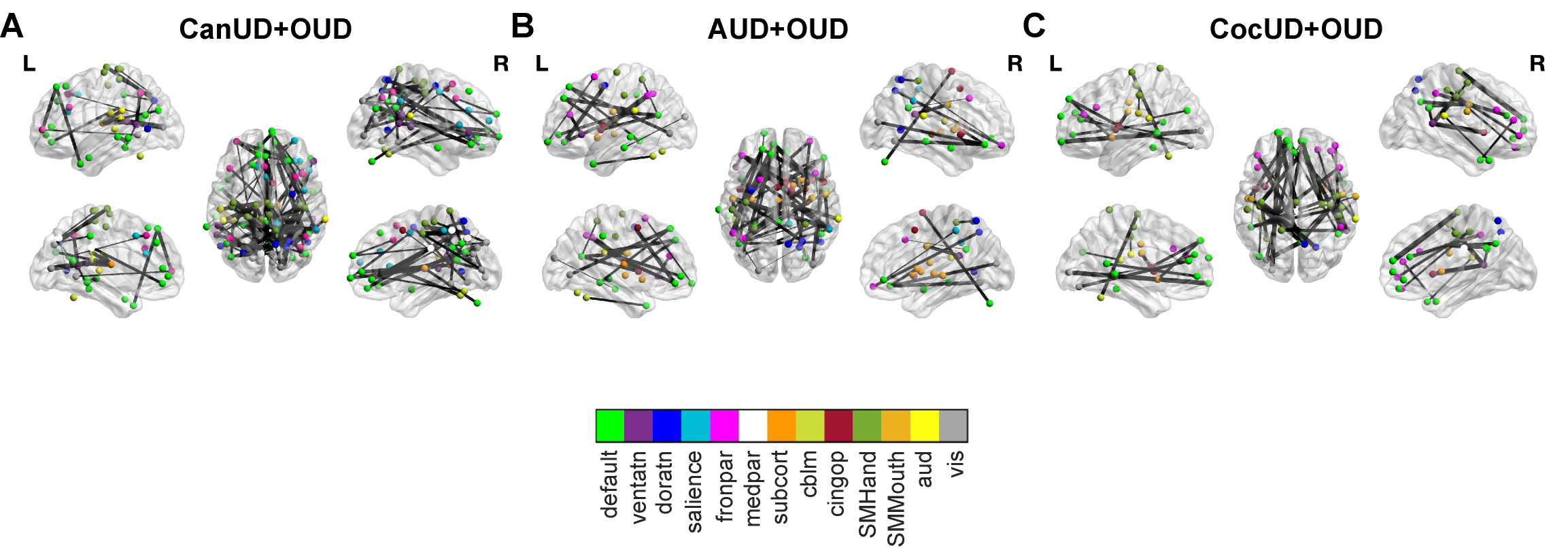

Supplement: Supplementary file 6 — Figure S6: Connections with highest TFNBS scores predominantly involve DMN across the conditions presented. ROIs from the nonsymmetric Power atlas are plotted on a template brain. Only the top 5% individual ROIs with the largest differences in TFNBS values pre‐ versus on‐NTX for each condition are visualized for clarity. ROIs are colour coded by network. Each black bar is weighted to represent the magnitude of difference in Pearson correlation value for each connection pre‐ to on‐NTX for (A) CanUD+OUD, (B) AUD + OUD and (C) CocUD+OUD. [file ADB-31-e70159-s004.BMP]

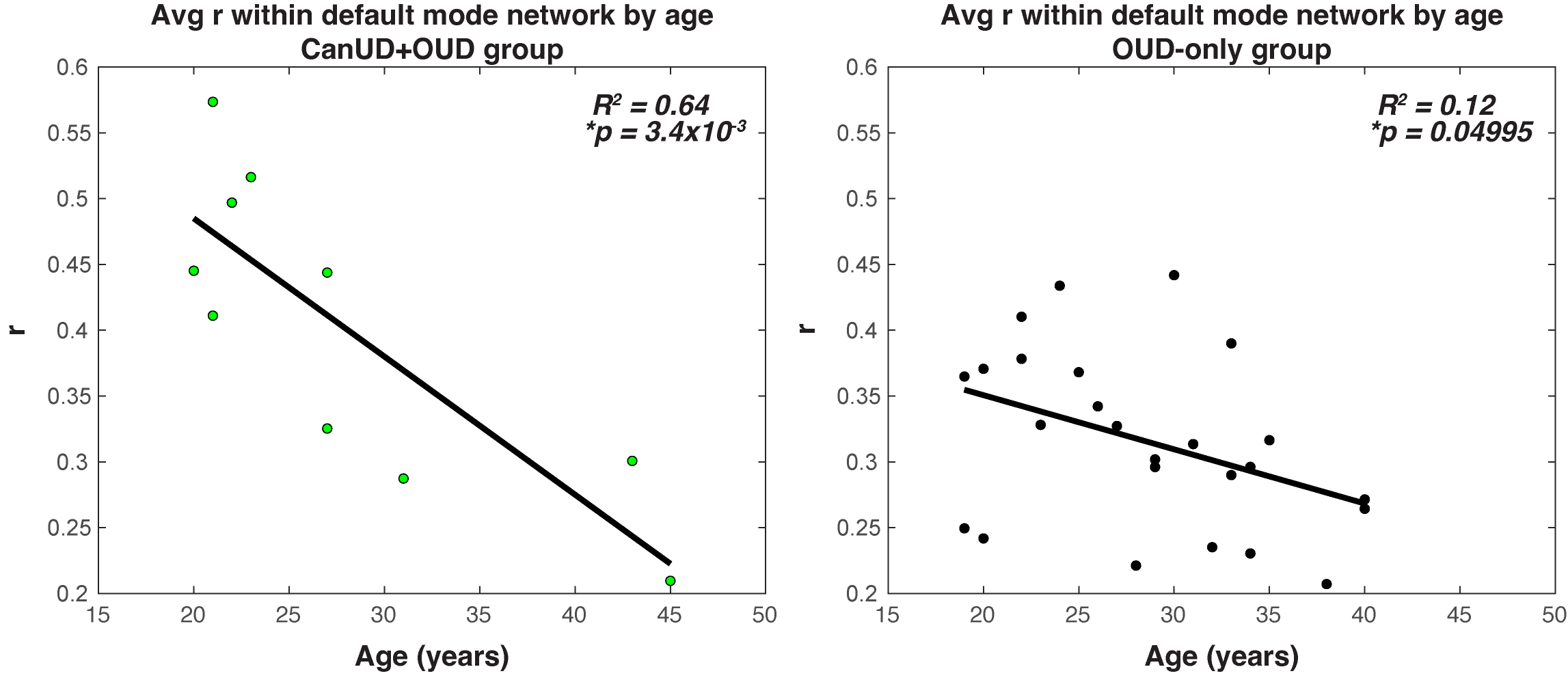

Supplement: Supplementary file 7 — Figure S7: In default mode network, younger subjects had bigger FC alterations at baseline in those with CanUD+OUD. Linear regression between average Pearson correlation coefficient (r) within default mode network and subject age in (Left) CanUD+OUD and (Right) OUD‐only. [file ADB-31-e70159-s005.BMP]
